# A Universal Vacant Parking Slot Recognition System Using Sensors Mounted on Off-the-Shelf Vehicles

**DOI:** 10.3390/s18041213

**Published:** 2018-04-16

**Authors:** Jae Kyu Suhr, Ho Gi Jung

**Affiliations:** 1 School of Intelligent Mechatronics Engineering, Sejong University, 209 Neungdong-ro, Gwangjin-gu, Seoul 05006, Korea; jksuhr@sejong.ac.kr; 2 Department of Electronic Engineering, Korea National University of Transportation, 50 Daehak-ro, Chungju-si, Chungbuk 27469, Korea

**Keywords:** automatic parking system, parking slot recognition, around-view monitor (AVM) system, sensor fusion

## Abstract

An automatic parking system is an essential part of autonomous driving, and it starts by recognizing vacant parking spaces. This paper proposes a method that can recognize various types of parking slot markings in a variety of lighting conditions including daytime, nighttime, and underground. The proposed method can readily be commercialized since it uses only those sensors already mounted on off-the-shelf vehicles: an around-view monitor (AVM) system, ultrasonic sensors, and in-vehicle motion sensors. This method first detects separating lines by extracting parallel line pairs from AVM images. Parking slot candidates are generated by pairing separating lines based on the geometric constraints of the parking slot. These candidates are confirmed by recognizing their entrance positions using line and corner features and classifying their occupancies using ultrasonic sensors. For more reliable recognition, this method uses the separating lines and parking slots not only found in the current image but also found in previous images by tracking their positions using the in-vehicle motion-sensor-based vehicle odometry. The proposed method was quantitatively evaluated using a dataset obtained during the day, night, and underground, and it outperformed previous methods by showing a 95.24% recall and a 97.64% precision.

## 1. Introduction

As the demand for autonomous driving has rapidly increased, automatic parking systems have also been actively researched [1,2]. Automatic parking systems start by recognizing vacant parking spaces, and the methods for this task can be categorized into four approaches: user-interface-based, free-space-based, slot-marking-based, and infrastructure-based [3]. Among them, the free-space-based and slot-marking-based approaches have been more extensively developed in the automobile industry because they can be fully automated and implemented only with vehicle-mounted sensors. The free-space-based approach finds vacant parking spaces by recognizing adjacent vehicles. This approach works well in situations where the positions of the adjacent vehicles are favorable. However, it cannot find free spaces when there is no adjacent vehicle and its accuracy depends on the positions of adjacent vehicles. The slot-marking-based approach can mitigate this drawback. Since this approach finds parking spaces by recognizing markings on road surfaces, it can accurately localize parking spaces regardless of the existence and positions of adjacent vehicles in the presence of parking slot markings.

**Figure 1:**
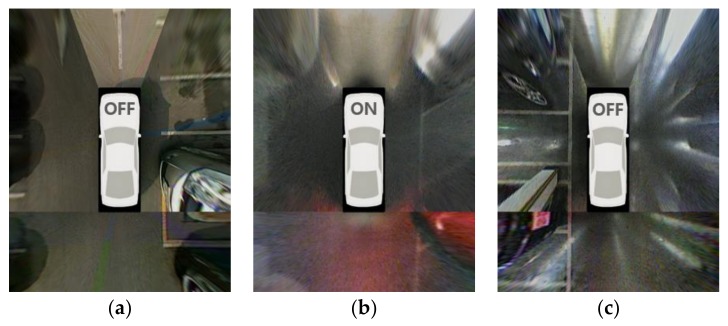
Parking lot images taken by an around-view monitoring (AVM) system. (**a**) Daytime; (**b**) Nighttime; (**c**) Underground.

In order for a slot-marking-based method to be useful and practical, it needs to satisfy the following conditions: it is able to be fully automated, handle various types of parking slot markings, and operate in a variety of lighting conditions. Since almost all slot-marking-based methods use cameras, it is particularly important that reliable performances can be achieved at night and underground, where severe lighting conditions are present. Figure 1 shows three parking lot images taken by an around-view monitoring (AVM) system in daytime, nighttime, and underground settings. In this figure, it can be seen that lighting conditions are quite unfavorable at night and underground due to dim lighting, amplified noise, and reflections on the road surface. These images are from the test dataset. The ON/OFF indication on the vehicle bonnet indicates whether the headlights are operating.

Regarding the abovementioned conditions of the slot-marking-based approach, the previous methods can briefly be summarized as follows: The first method to recognize various types of parking slot markings was proposed in [3]. This method could not be fully automated because a driver has to manually designate the position of the parking slot’s entrance. The method in [4] overcame this limitation by automatically detecting various types of parking slot markings using a hierarchical tree structure. However, since this method is sensitive to lighting conditions, it can only be applied under daytime conditions. To complement this method, the method in [5] was suggested. This method can reliably operate in underground parking lots where lighting conditions are unfavorable, but it can only handle a single type of parking slot marking. A detailed literature review will be presented in Section 2.

Therefore, in order to overcome the limitations of these previous methods, this paper proposes a method that can operate in a fully automatic manner, recognize various types of parking slot markings, and achieve robustness against a variety of lighting conditions. The proposed method works as follows: This method first detects the separating lines of parking slot markings by extracting parallel line pairs from AVM images. The separating lines detected in the current image are then combined with those detected in the previous images. Parking slot candidates are generated by pairing the combined separating lines based on the geometric constraints of the parking slot. These candidates are confirmed by recognizing their entrance positions using both line and corner features and classifying their occupancies using ultrasonic sensors. Finally, the vacant parking slots recognized in the current image are combined with those recognized in the previous images. The current positions of the separating lines and parking slots found in the previous images are tracked based on the in-vehicle motion sensor-based vehicle odometry. The proposed method was quantitatively evaluated with the dataset obtained in various conditions including daytime, nighttime, and underground. This method outperformed the previous methods by showing a 95.24% recall and a 97.64% precision.

The contributions of this paper are as follows:

(1) To recognize various types of parking slot markings with open and close entrances, this paper proposes an approach that generates parking slot candidates based on separating lines and confirms their entrances using both line and corner features. Previous parallel line-based methods cannot handle parking slot markings with open entrances because they only detect line features.
(2) To achieve robustness in severe lighting conditions at night and underground, this paper proposes an approach that tracks separating lines and parking slots using in-vehicle motion sensor-based odometry, whose accuracy is independent of lighting conditions. Previous slot-marking-based methods have limitations in handling severe lighting conditions because they only use features extracted from the current image.
(3) This paper efficiently combines the two aforementioned approaches to develop a universal parking slot recognition method that can handle various types of parking slot markings during the day, at night, and underground. The proposed method was quantitatively evaluated using the dataset obtained under various severe lighting conditions and showed outstanding performance. According to our literature review, there has been no previous method that can handle various types of parking slot markings and a variety of severe lighting conditions, and no previous method has been quantitatively evaluated in these different settings.

The rest of this paper is organized as follows: Section 2 explains the related research. Section 3 and Section 4 introduce the sensor configuration and overview of the proposed method, respectively. Section 5 describes the separating line detection and tracking. Section 6 and Section 7 explain the parking slot generation, tracking, and combining, respectively. Section 8 presents the experimental results. The paper is concluded in Section 9.

## 2. Related Research

The parking space detection methods can be categorized into four approaches: user-interface-based, free-space-based, slot-marking-based, and infrastructure-based. The literature review of this paper focuses on the first three approaches that can be implemented with only the vehicle-mounted sensors.

### 2.1. User-Interface-Based Approach

The user-interface-based approach finds vacant parking spaces based on the driver’s manual input. Even though the methods in this approach cannot be automated, they are useful as backup tools for automatic methods. The method in [6] interacts with a driver using an augmented display. It helps parallel parking by allowing a driver to locate a vertical pole at the end of the parked vehicle. The method in [7] utilizes an arrow button-based interface. A driver can locate a rectangle at the desired parking space by clicking several arrow buttons. This method has been applied to the mass-produced Toyota Prius [8]. The method in [9] uses a drag-and-drop interface. A desired parking space can be manually located by dragging the inside and outside of a rectangle displayed in a touch screen.

### 2.2. Free-Space-Based Approach

The free-space-based approach recognizes vacant parking spaces by finding adjacent parked vehicles. This is the most popular approach since various range sensors can be used to find adjacent parked vehicles. However, this approach has a fundamental drawback in that it cannot properly work when there is no adjacent parked vehicle, so its performance is highly dependent on the positions of the adjacent parked vehicles. Among various range sensors, ultrasonic sensors are most popularly used because they are relatively inexpensive and easy to mount on mass-produced vehicles. The ultrasonic-sensor-based method has been adopted by most car manufacturers [10,11,12,13]. It recognizes vacant parking spaces based on the range data acquired by registering the outputs of the ultrasonic sensors. Cameras have also been popularly used in this approach. Most of the camera-based methods recognize vacant parking spaces by reconstructing surrounding 3D structures. The 3D reconstruction has been conducted by a variety of techniques including monocular motion stereo [14,15,16], binocular stereo [17,18], and structured light [19]. Contrary to these methods, the method in [20] recognizes vacant parking spaces based on the horizontal edges of the adjacent vehicles without 3D reconstruction. The camera-based methods can readily use the cameras already mounted on mass-produced vehicles, but they are sensitive to lighting conditions and usually require a large amount of computation for 3D reconstruction. The method in [21] fuses a camera and ultrasonic sensors in a low-level. It recognizes vacant parking spaces by combining the range data obtained from monocular motion stereo and ultrasonic sensors. Laser scanners have also been used for this approach and produced excellent performances because their range data are highly accurate [22,23]. However, laser scanners have drawbacks in that they are costly and have a durability problem due to rotation mirrors. Besides these sensors, photonic mixer devices [24] and short-range radars [25,26,27] have also been used in this approach.

### 2.3. Slot-Marking-Based Approach

The slot-marking-based approach recognizes vacant parking spaces by analyzing markings on road surfaces. Contrary to the free-space-based approach, its performance is not dependent on the existence and positions of adjacent parked vehicles. However, in order for this approach to properly work, parking slot markings should be presented. All methods in this approach use cameras that can easily capture markings on the ground. These methods can be categorized into semi-automatic and fully automatic. The methods in [3,28,29] detect parking slot markings in a semi-automatic manner. The one-touch method in [28] recognizes parking slot markings based on one manually designated point, and an efficient version of this method was presented in [29]. Since this method can deal with only a single type of parking slot marking, the two-touch method in [3] was proposed. It can recognize various types of parking slot markings based on two manually designated points. Unlike these semi-automatic methods, the methods in [4,5,30,31,32,33,34,35,36,37,38,39,40,41] detect parking slot markings in a fully automatic manner. The method in [30] recognizes parking slots based on neural-network-based color segmentation. The method in [31] detects parallel line pairs using a specialized filter and Hough transform to recognize parking slot markings. The method in [32] uses a similar approach that detects parallel line pairs using Radon transform. The method in [33] detects straight lines using an improved random sample consensus (RANSAC) algorithm and recognizes parking slots based on the line detection result. The method in [34] recognizes parking slots using vertically oriented lines and their occupancies using a difference-of-Gaussians-based histogram. The method in [35] extracts parking slot boundaries by applying a sequential RANSAC line estimator to binarized ridge images. The method in [36] detects parallel line pairs and lines perpendicular to them using a probabilistic Hough transform and recognizes parking slots by combining those lines. The method in [5] reliably recognizes parking slots in indoor and underground settings by detecting a guideline using RANSAC and separating lines using distance transform. The method in [37] detects junctions of parking slots based on adaptive boosting with integral channel features and recognizes parking slots by combining the detected junctions. While all of the above methods have a limitation in that they can only deal with one or two types of parking slot markings, several methods have been proposed to handle more types of parking slot markings. The method in [38] recognizes various types of parking slot markings using a hierarchical tree structure in rearview camera images. This method has been applied to AVM images in [39] and combined with an ultrasonic-sensor-based occupancy classification in [4]. The method in [40] clusters a con-hat filtering result and detects lines using RANSAC to recognize parking slots in rearview camera images, and this method has been applied to AVM images in [41]. It works well in the case of parking slots with closed entrances but cannot recognize those with open entrances. In terms of lighting conditions, there has been no previous method that quantitatively shows its ability to handle various types of parking slot markings under unfavorable lighting conditions such as nighttime and underground.

The proposed method can be categorized as a slot-marking-based approach. Compared with the other previous methods in this approach, this method can operate in a fully automatic manner without any driver intervention, recognize various types of parking slot markings whether its entrance is close or open, and reliably work even in nighttime and underground settings.

## 3. Sensor Configuration

**Figure 2:**
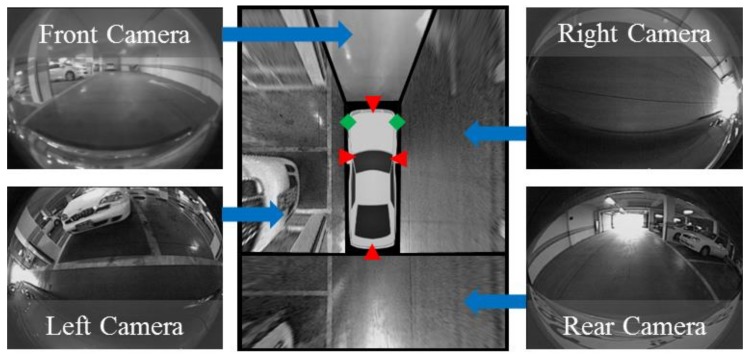
Sensor configuration. Red triangles and green diamonds are four cameras of the AVM system and two ultrasonic sensors, respectively.

The proposed method uses only those sensors already mounted on an off-the-shelf vehicle, the Hyundai Azera [42]. They include an AVM system, ultrasonic sensors, and in-vehicle motion sensors. The AVM system is composed of four fisheye cameras located at the centers of the front and rear bumpers and under the side-view mirrors as shown in Figure 2 with red triangles. Four images taken from four different cameras are transformed into bird’s-eye-view images and are stitched to each other in order to produce an AVM image. The resolution of each fisheye camera is 640 × 480 pixels and that of the AVM image is 360 × 480 pixels. Two ultrasonic sensors are located at both sides of the front bumper as shown in Figure 2 with green diamonds. Their operating range and resolution are 30~450 cm and 2 cm, respectively. In-vehicle motion sensors including wheel speed and yaw rate sensors are located inside the vehicle, and are used for vehicle odometry estimation. The vehicle odometry is calculated by the parking assistant system embedded in the Hyundai Azera, and the proposed method uses odometry calculation results obtained through the in-vehicle network. It contains the orientation angle and displacements in longitudinal and lateral directions with respect to the center of the two rear wheels. The resolutions of the orientation angle and displacement are 0.1° and 0.1 cm, respectively. All those data are synchronized to have a 15 Hz acquisition frequency.

## 4. Overview of the Proposed Method

**Figure 3:**
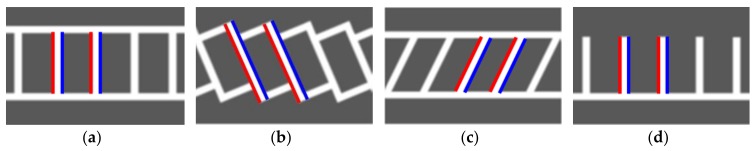
Four types of parking slot markings. (**a**) Rectangular type; (**b**) Slanted rectangular type; (**c**) Diamond type; (**d**) Open rectangular type.

The proposed method utilizes a line-detection-based approach for recognizing parking slots because this approach has been proven to be more robust against lighting conditions compared with the corner-based approach [5]. This method handles four types of parking slot markings: rectangular, slanted rectangular, diamond, and open rectangular as shown in Figure 3. In this figure, a line marking composed of a pair of red and blue lines is called a separating line because it separates adjacent parking slots. Since the parking slots of various types consist of separating line pairs, the proposed method recognizes parking slots using an approach that detects and combines separating lines. This method also tracks the detected separating lines through the image sequence using the vehicle odometry. This approach can increase the recognition performance because it is difficult to detect the separating lines in every image, especially in severe lighting conditions. In addition, this method uses both line and corner features when recognizing the parking slots’ entrances. This approach makes it possible to handle parking slots with both closed and open entrances.

**Figure 4:**
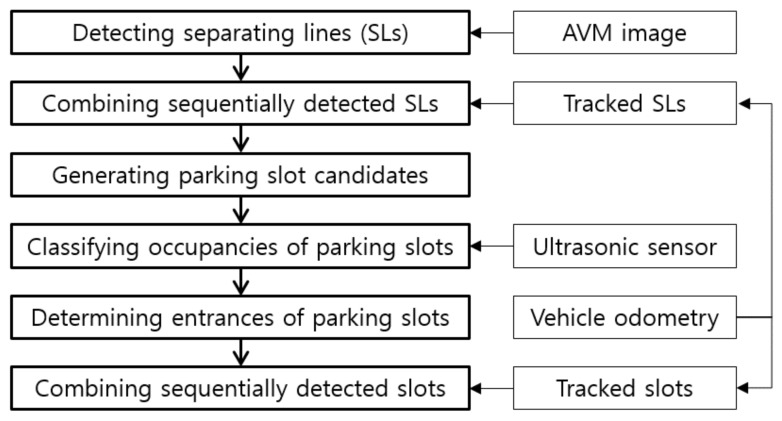
Flowchart of the proposed method.

Figure 4 shows the flowchart of the proposed method. This method first detects separating lines from an AVM image. The detected separating lines are then combined with the previously detected separating lines whose positions are tracked by the vehicle odometry. Once the separating lines are combined, parking slot candidates are generated by pairing the separating lines based on the geometric constraints of the parking slot. The parking slot candidates are then confirmed by the ultrasonic-sensor-based occupancy classification followed by the line- and corner-feature-based entrance determination. Finally, the recognized vacant parking slots are combined with the previously recognized vacant parking slots whose positions are tracked by the vehicle odometry in order to achieve a more reliable recognition result.

## 5. Separating Line Detection and Tracking

### 5.1. Separating Line Detection

The separating line has two important properties: one is that it consists of two parallel lines and the other is that gradient directions of two parallel lines are opposite to each other. Red and blue line pairs in Figure 3 indicate the line pairs constituting the separating lines. It can be noticed that the red and blue lines are parallel and their gradient directions are opposite. The proposed method detects the separating lines based on these two properties. One of the simplest ways to find parallel line pairs is to detect multiple lines and then pair them together. However, this approach has limitations in that it needs a sophisticated line pairing condition and reduces robustness by separately handling two correlated lines. Thus, the proposed method uses an approach that simultaneously finds a parallel line pair whose gradient orientations are opposite. The methods in [31] and [32] also find parallel lines using Hough and Radon transforms, respectively. However, they ignore one of the important properties that gradient detections of two lines are opposite and suffer from the problem of parameter resolution, a fundamental limitation of the voting-based approach. Therefore, the proposed method utilizes RANSAC in order to accurately and robustly detect parallel line pairs.

The Sobel operator [43] is first applied to the AVM image for extracting edge pixels and their gradient orientations, and RANSAC is then applied to those edge pixels. To properly apply RANSAC, a least squares estimator should be formulated in advance. A pair of parallel lines can be described as

(1) $$\begin{array}{l} \alpha u+\beta =v\\ \alpha u^{\prime }+\gamma =v^{\prime } \end{array}$$

where *α*, *β*, and *γ* are parameters of a pair of parallel lines, and (*u*, *v*) and (*u*′, *v*′) are locations of the edge pixels whose gradient orientations are opposite. If two parallel lines are composed of *N* and *M* edge pixels, Equation (1) can be rephrased as

(2) $$\underset{A}{\underset{︸}{\left[\begin{array}{ccc} u_{1} & 1 & 0\\ \vdots & \vdots & \vdots \\ u_{N} & 1 & 0\\ u_{1}^{\prime } & 0 & 1\\ \vdots & \vdots & \vdots \\ u_{M}^{\prime } & 0 & 1 \end{array}\right]}}\underset{x}{\underset{︸}{\left[\begin{array}{c} \alpha \\ \beta \\ \gamma \end{array}\right]}}=\underset{b}{\underset{︸}{\left[\begin{array}{c} v_{1}\\ \vdots \\ v_{N}\\ v_{1}^{\prime }\\ \vdots \\ v_{M}^{\prime } \end{array}\right]}}.$$

**x**, which minimizes the least squares error, can be estimated by the pseudo-inverse as

(3) $$x={\left(A^{T}A\right)}^{-1}A^{T}b.$$

The proposed method applies RANSAC to the above least squares estimator as follows:

1. Randomly select one edge pixel.
2. Randomly select two edge pixels whose orientations are opposite to that of the previously selected one.
3. Estimate parallel line pair parameters using the selected three edge pixels via the least squares estimator.
4. Count consensus sets (edge pixels) based on perpendicular distance. Orientations of the consensus sets should coincide with those of the randomly selected edge pixels.
5. Repeat the above procedures and select the parameters that give the maximum number of consensus set.
6. Re-estimate parallel line pair parameters using all edge pixels in the selected consensus set based on the least squares estimator.

**Figure 5:**
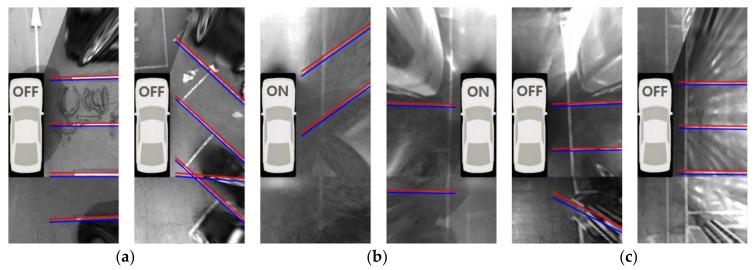
Separating line detection results. (**a**) Daytime; (**b**) Nighttime; (**c**) Underground.

RANSAC needs a threshold value that determines whether an edge pixel is an inlier or not. This value is experimentally set to 2 pixels. It means that the separating line detection method allows edge pixels deviating by ±2 pixels from the ideal line. To detect multiple parallel line pairs, the proposed method repeats the above procedure while removing the edge pixels that support the previously estimated line pairs. This method discards the separating line if its width is dissimilar to the prior knowledge of the marking line width (15~25 cm) or it is not supported by enough edge pixels. In most cases, the separating lines are not parallel to the travel direction of the vehicle when the vehicle passes beside the parking slots. Thus, the separating lines whose directions are similar to the travel direction of the vehicle are discarded. Figure 5a–c show the separating lines detected by the proposed method in daytime, nighttime, and underground settings, respectively. In this figure, the area of the AVM image with no parking slot is omitted for conciseness. Note that, in Figure 5, the separating line is drawn from the image boundary to the position near the side of the vehicle because the separating line detection method does not explicitly recognize the start and end positions. It can be found that the proposed method can reliably find multiple separating lines regardless of lighting conditions. Since the separating lines whose directions are similar to the travel direction of the vehicle are removed, most of the entrance lines are not detected during the separating line detection procedure. Missed separating lines will be substituted while sequential detection results are combined, and falsely detected separating lines will be removed while parking slot candidates are generated. This will be explained later in detail. This paper assumes that the AVM system is properly calibrated at its manufacturing stage. If the AVM system is incorrectly calibrated, the performance of the parallel line detection may be degraded.

### 5.2. Separating Line Tracking

**Figure 6:**
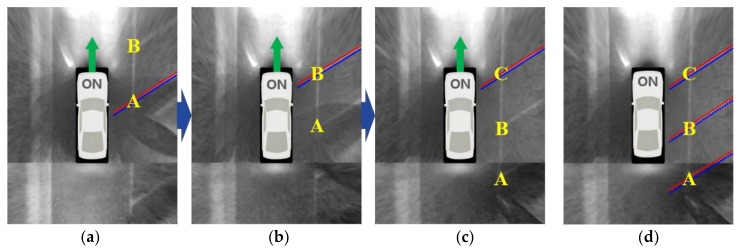
Separating line detection and tracking at night. (**a**–**c**) The consecutive detection results using only the current image; (**d**) The detection result obtained by combining the consecutive detection results via the vehicle-odometry-based tracking.

Under severe lighting conditions, the separating line detection performance is normally degraded. For instance, at night, a separating line near the camera is relatively well detected, but a separating line far from the camera is hardly detected because the image quality of that area is quite inferior. Figure 6a–c show AVM images taken while the vehicle is moving next to the parking slots at night. In these figures, A, B, and C are indexes of three separating lines. In Figure 6a, A is detected because it is located near the camera, but B is not detected due to the inferior image quality. In Figure 6b, as the vehicle moves, B becomes close to the camera and is detected, but A becomes further from the camera and is not detected. In Figure 6c, as the vehicle moves again, C is detected but A and B are not detected. In such a situation, parking slots cannot be reliably recognized if only the separating lines detected in the current image are used. Thus, the proposed method utilizes an approach that tracks positions of the separating lines detected in previous images and recognizes parking slots using the separating lines detected in both the current and previous images. The separating lines are tracked based on the vehicle odometry calculated by in-vehicle motion sensors: wheel speed and yaw rate sensors. These sensors have been already mounted on mass-produced vehicles. When the orientation angle and displacement vector of the vehicle are *θ* and [*t_x_ t_y_*]^T^, respectively, and the parameters of the previously detected line is **l***_p_* = [*α* –1 *β*]^T^, where α and β are the line parameters shown in Equation (1), the tracked parameters of the corresponding line in the current image, **l***_c_* can be calculated as

(4) $$l_{c}=H^{-T}l_{p},\;where\;H=\left[\begin{array}{ccc} \cos\theta & -\sin\theta & t_{x}\\ \sin\theta & \cos\theta & t_{y}\\ 0 & 0 & 1 \end{array}\right].$$

If the currently detected separating line overlaps the previously detected and tracked separating line, the one supported by a larger number of edge pixels is selected. When comparing those numbers, the number for the tracked separating line is weighted by *r* (<1.0) because its position includes a cumulative error induced by the vehicle odometry. *r* is experimentally set to 0.7. Whether or not the two lines overlap is determined based on the similarity of the slope and y-intercept. Figure 6d shows the separating lines obtained by combining those detected in both the current and previous images. In this figure, C is detected in the current image, while A and B are detected in the previous images and tracked based on the vehicle odometry. If this approach is applied, the parking slot recognition performance is expected to increase because it is possible to use the separating lines located at both the superior and inferior quality regions of the current image.

## 6. Parking Slot Generation

### 6.1. Parking Slot Candidate Generation

**Figure 7:**
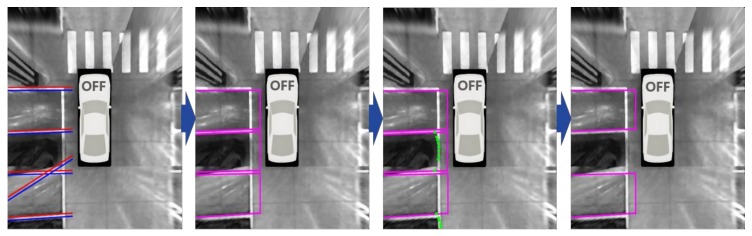

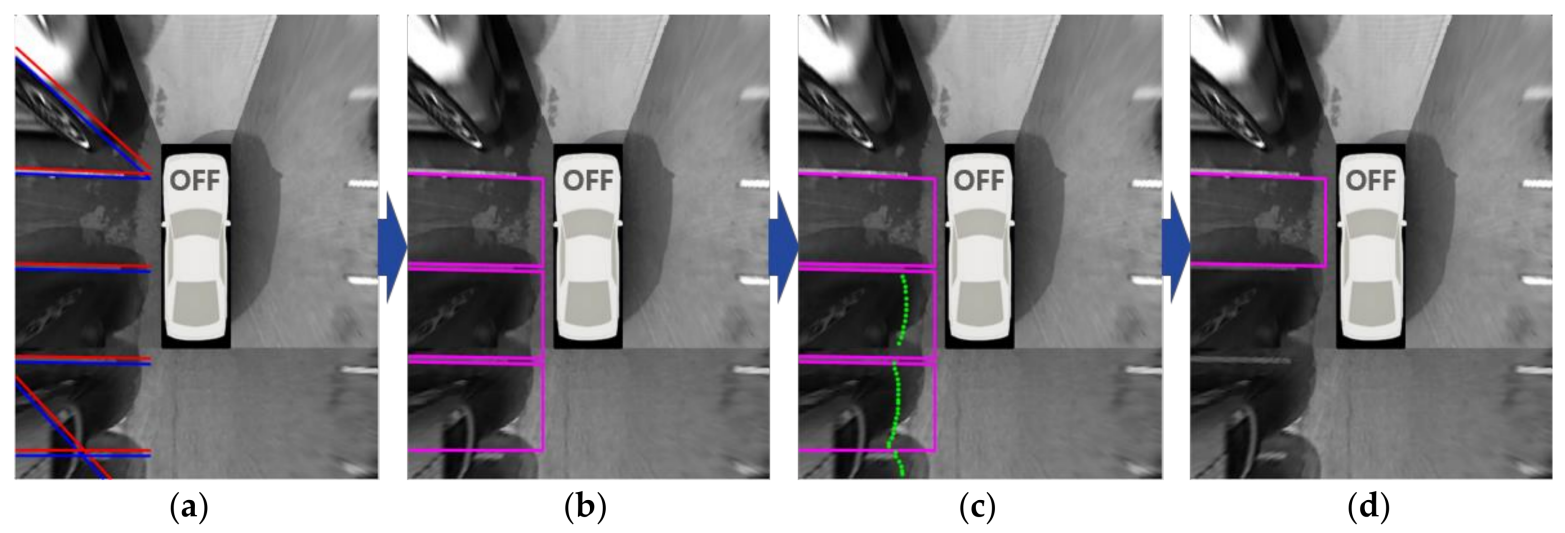
Parking slot candidate generation and occupancy classification. (**a**) Separating line detection results; (**b**) Parking slot candidate generation results; (**c**) Range data obtained by an ultrasonic sensor; (**d**) Parking slot candidates classified as vacant.

Parking slot candidates are generated by pairing the separating lines based on the geometric constraints of the parking slot. The proposed method uses two simple geometric constraints: two separating lines are parallel, and the width between two separating lines is in a certain range (190~350 cm). Although the separating line tracking can increase the detection rate, it might increase the number of false detections. Thus, in order to prevent false parking slot candidates, the proposed method uses an additional constraint that at least one separating line should be detected in the current image. Figure 7a,b depict the parking slot candidate generation procedure. In these figures, the first row shows the rectangular type parking slots that have closed entrances, and the second row shows the open rectangular type parking slots that have open entrances. Figure 7a depicts the separating line detection results, and Figure 7b depicts the parking slot candidates generated by the separating lines in Figure 7a. It can be seen that the parking slot candidates are correctly generated, while some falsely detected separating lines that do not follow the aforementioned geometric constraints are discarded. Note that the entrances of the parking slots are not yet recognized, so they are arbitrarily drawn in this figure. The procedure for the entrance recognition will be explained in Section 6.3.

### 6.2. Parking Slot Occupancy Classification

After generating the parking slot candidates, occupancies of those candidates are classified. If a slot candidate is determined as occupied, this slot is deleted because the vehicle cannot park in it. The occupancy classification is conducted based on the range data acquired by ultrasonic sensors. Two ultrasonic sensors are located at both sides of the front bumper as shown in Figure 2 with green diamonds. Green dots in Figure 7c indicate the range data obtained by registering the outputs of the ultrasonic sensor while the vehicle is moving. This paper does not propose a parking slot occupancy classification method but adopts the method proposed in [4]. This method treats a parking slot as a single cell of the occupancy grid and calculates the posterior probability to be occupied. In this case, the occupancy of the parking slot can be modeled as a binary estimation problem with static state [44]. The posterior probability of parking slot occupancy can be sequentially calculated in log odds representation as

(5) $$\begin{array}{ll} l_{1:t}\left(O\right) & =\log\frac{p\left(O|z_{1:t}\right)}{1-p\left(O|z_{1:t}\right)}=\log\left\{\frac{p\left(O|z_{t}\right)}{1-p\left(O|z_{t}\right)}\cdot \frac{p\left(O|z_{1:t-1}\right)}{1-p\left(O|z_{1:t-1}\right)}\cdot \frac{1-p\left(O\right)}{p\left(O\right)}\right\}\\ & =\log\frac{p\left(O|z_{t}\right)}{1-p\left(O|z_{t}\right)}+\log\frac{p\left(O|z_{1:t-1}\right)}{1-p\left(O|z_{1:t-1}\right)}-\log\frac{p\left(O\right)}{1-p\left(O\right)}\\ & =l_{t}\left(O\right)+l_{1:t-1}\left(O\right)-l_{o}\left(O\right) \end{array}$$

where *O* means the parking slot is occupied, and *V* has the opposite meaning. *z_t_* represents whether the ultrasonic sensor output at time index *t* is located inside the parking slot candidate, and it is either positive (*P*) or negative (*N*). *p*(*O*|*z*_1:*t*_) is a posterior probability to be occupied given the ultrasonic sensor outputs from time index 1 to *t*. *p*(*O*) is a prior probability to be occupied and is set to 0.5. To utilize Equation (5), *p*(*O*|*P*) and *p*(*O*|*N*), two cases of *p*(*O*|*z_t_*) were pre-calculated by Bayes’ theorem using *p*(*P*|*O*) and *p*(*P*|*V*), which were estimated based on the training dataset. The training dataset includes 456 occupied and 357 vacant parking slots of various types with the corresponding ultrasonic sensor outputs. This training dataset is totally different from the test dataset. The log odds ratio in Equation (5) can be converted to the posterior probability as

(6) $$p\left(O|z_{1:t}\right)=1-\frac{1}{1+\exp\left\{l_{1:t}\left(O\right)\right\}}.$$

Occupancies of the parking slot candidates are classified based on the posterior probabilities, *p*(*O*|*z*_1:*t*_), and those classified as occupied are removed. Figure 7d shows the parking slot candidates classified as vacant using the range data shown in Figure 7c.

### 6.3. Parking Slot Entrance Detection

**Figure 8:**
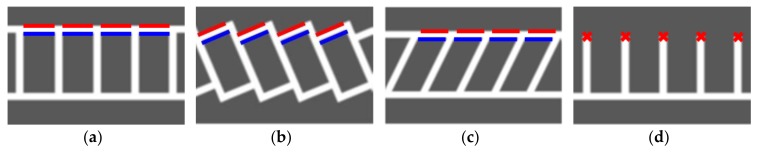
Entrances of four types of parking slot markings. (**a**) Rectangular type; (**b**) Slanted rectangular type; (**c**) Diamond type; (**d**) Open rectangular type.

Once the vacant parking slot candidates are selected, their entrances are found. To this end, the proposed method uses both line- and corner-based approaches. Figure 8 shows the shapes of the entrances for four types of parking slot markings. Since the entrances of the rectangular, slanted rectangular, and diamond types are composed of lines, the proposed method utilizes the line-based approach. However, the entrance of the open rectangular type cannot be found by the same approach because its entrance does not consist of lines. Thus, the corner-based approach is utilized in this case. The proposed method first applies the line-based approach to the vacant parking slot candidate. If this approach cannot find the entrance, the corner-based approach is then applied.

**Figure 9:**
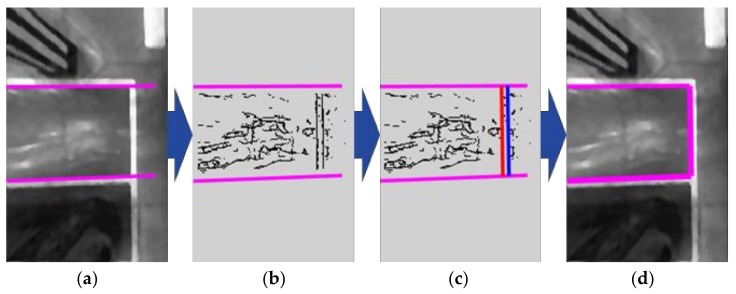
Line-based entrance detection procedure. (**a**) Separating line pair constituting a parking slot candidate; (**b**) Edge pixels between two separating lines; (**c**) RANSAC-based parallel line pair detection result; (**d**) Completely recognized parking slot.

The line-based entrance detection is almost the same as the parallel line pair detection used for finding the separating lines in Section 5.1. In this case, only the edge pixels located between two separating lines constituting the parking slot candidate is used. The edge pixels already found during the separating line detection are reused. Figure 9 depicts the line-based entrance detection procedure. Figure 9a shows a separating line pair constituting a parking slot candidate of rectangular type and Figure 9b shows the edge pixels located between two separating lines. A pair of red and blue lines in Figure 9c indicates the parallel line pair detection result produced by RANSAC, and Figure 9d presents the completely recognized parking slot after the line-based entrance detection.

If the number of edge pixels classified as inliers by the line-based entrance detection using RANSAC is smaller than a predetermined value (25% of the slot width), the parking slot candidate is considered as an open rectangular type, and the corner-based entrance detection is then applied. The corner-based entrance detection is based on cornerness values on two separating lines constituting a parking slot candidate. The cornerness value indicates how much an image location is similar to the corner and is calculated based on the second moment matrix used for Harris corner detector [45]. The second moment matrix can be expressed as

(7) $$M=\sum_{\left(u,v\right)\in W}\left[\begin{array}{cc} I_{u}^{2} & I_{u}I_{v}\\ I_{u}I_{v} & I_{v}^{2} \end{array}\right]$$

where (*u*, *v*) indicates the image location, and *W* is a small spatial neighborhood around (*u*, *v*). *I_u_* and *I_v_* are the image gradients in the horizontal and vertical directions, respectively. Since the matrix *M* contains information on the brightness variation in *W*, the image location (*u*, *v*) is considered as a corner if both eigenvalues (*λ*_1_, *λ*_2_) of *M* are large. Thus, the smaller eigenvalue (*λ_min_*) of *λ*_1_ and *λ*_2_ is used for the cornerness value. *λ_min_* can be efficiently calculated by the following approximation [45]:

(8) $$\lambda_{min}\approx \frac{\lambda_{1}\lambda_{2}}{\left(\lambda_{1}+\lambda_{2}\right)}=\frac{det\left(M\right)}{trace\left(M\right)}.$$

**Figure 10:**
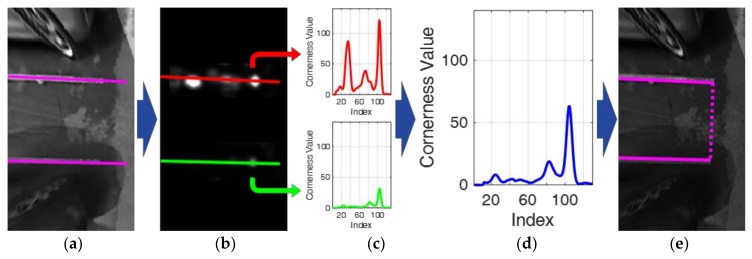
Corner-based entrance detection procedure. (**a**) Separating line pair constituting a parking slot candidate; (**b**) Cornerness values; (**c**) Two cornerness value profiles extracted from two separating lines; (**d**) Combined cornerness value profile; (**e**) Completely recognized parking slot.

Figure 10 depicts the corner-based entrance detection procedure. Figure 10a shows a separating line pair constituting a parking slot candidate of open rectangular type, and Figure 10b shows the cornerness values on the separating line pair. It can be noticed that the entrance positions, which are the ends of the separating lines have high cornerness values. Once the cornerness values are calculated, a cornerness value profile is extracted from each separating line. Figure 10c shows two cornerness value profiles (*C*_1_ and *C*_2_) extracted from two separating lines. *C*_1_ and *C*_2_ are combined as

(9) $$C=\left(C_{1}+C_{2}\right)-\left|C_{1}-C_{2}\right|.$$

The sum of two profiles is subtracted by the difference of the two profiles because it is desired that the location where two profiles both have large cornerness values is found. Discarding the location where the cornerness value is large on only one profile can reduce false entrance detection. If the maximum value of C is smaller than a predetermined value (50), the parking slot candidate is not confirmed as a parking slot and rejected. Figure 10d shows the combined cornerness value profile, *C*, calculated by *C*_1_ and *C*_2_ in Figure 10c. In this figure, the horizontal axis (index) refers to the coordinates moved by 1 pixel along the separating line. The location where *C* is maximized is detected as the entrance location. Figure 10e presents the completely recognized parking slot after the corner-based entrance detection. In the case of the open rectangular type, the entrance is depicted with a dotted line.

## 7. Parking Slot Tracking and Combining

The proposed method uses vacant parking slots recognized not only from the current image, but also from the previous images. Positions of the previously recognized parking slots are tracked based on the vehicle odometry. Since a parking slot consists of two separating lines and two entrance points, the current position of the previously recognized parking slot can be tracked by the matrix *H* in Equation (4). The separating line can be tracked using Equation (4), and the entrance point can be tracked as

(10) $$p_{c}=Hp_{p}$$

where **p***_p_* is the entrance point of the previous recognized parking slot and **p***_c_* is its tracked position.

Once the vacant parking slots recognized from the current and previous images are obtained, this method finally combines them to obtain more reliable results. The parking slot combination procedure starts by checking overlapping conditions of the obtained parking slots using the intersection of union (*IOU*). *IOU* between rectangular areas of two parking slots (*A_i_*, *A_j_*) is calculated as

(11) $$IOU(A_{i},\;A_{j})=\frac{\left|A_{i}\cap A_{j}\right|}{\left|A_{i}\cup A_{j}\right|}$$

The overlapping conditions can be categorized in two cases: one is the overlap between parking slots recognized from the current image, and the other is the overlap between parking slots recognized from the current and previous images. In the first case, only one of the two parking slots should be selected since two different parking slots are unable to overlap in real situations. Two parking slots are considered to overlap if *IOU* is larger than a predetermined value *T_c_*. In order to select a more appropriate parking slot, the proposed method uses two scores. The first score *s_l_* is based on the reliability of two separating lines constituting the parking slot and is calculated using the number of inlier edge pixels that support two separating lines as

(12) $$s_{l}=\min\left(1,\;\frac{N_{1}}{2L_{1}}\right)+\min\left(1,\;\frac{N_{2}}{2L_{2}}\right)$$

where *N*_1_ and *N*_2_ are the numbers of inlier edge pixels that support two separating lines, and *L*_1_ and *L*_2_ are the lengths of two separating lines in pixels. The reason for putting 2 in the denominator is that each separating line consists of two parallel lines. The minimum operator (min) is used to limit the maximum value to 1. The second score *s_e_* is calculated based on the reliability of the parking slot entrance. This score is calculated by the cornerness value in case of the open rectangular type and by the number of inlier edge pixels that supports the entrance in case of the other types as

(13) $$s_{e}=\{\begin{array}{cc} \min\left(1,\;\frac{K}{K_{\max}}\right), & if\;open\;rectangluar\\ \min\left(1,\;\frac{N_{3}}{2L_{3}}\right), & otherwise \end{array}$$

where *K* is the cornerness value of the parking slot entrance, which is calculated by finding the maximum value from the combined cornerness value profile in Figure 10d, and *K_max_* is the maximum value of *K*, which is experimentally predetermined. *N_3_* is the numbers of inlier edge pixels obtained by the line-based entrance detection procedure, and *L_3_* is the length of the parking slot entrance in pixels. The denominator includes 2 because the parking slot entrance consists of two parallel lines. If two parking slots recognized from the current image are determined to overlap based on *IOU* in Equation (11), the one that has a larger *s_c_* in Equation (14) is selected.

(14) $$s_{c}=s_{l}+s_{e}$$

**Figure 11:**
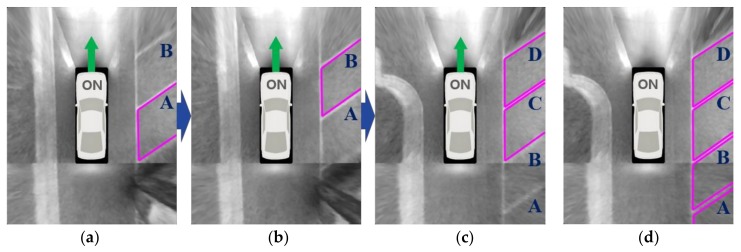
Parking slot tracking and combining procedure. (**a**–**c**) show the parking slot recognition results of three individual images; (**d**) shows the combined result of (**a**–**c**) using the suggested approach.

The second overlapping situation is the overlap between parking slots recognized from the current and previous images. This overlapping situation can be further categorized into two cases: one is that two parking slots almost completely overlap, and the other is that two parking slots slightly overlap. The first case is considered that the same parking slot in the real world is consecutively recognized in an image sequence. It is determined that this case occurs when *IOU* is greater than *T_s_* (≫*T_c_*). In this case, the number of repeated detections (*D*) for this parking slot is increased by 1, and the one that has the largest *s_c_* in Equation (14) is selected. When comparing these scores, *s_c_* of the previously recognized parking slot is weighted by *r* (<1.0) because its position includes a cumulative error induced by the vehicle odometry. *r* is experimentally set to 0.7. The second case is that two parking slots recognized from the current and previous images slightly overlap. It is determined that this case occurs when *IOU* is between *T_c_* and *T_s_*. Since two different parking slots are unable to overlap in real situations, the one that has a larger *s_t_* in Equation (15) is selected.

(15) $$s_{t}=s_{l}+s_{e}+s_{d},\;where\;s_{d}=\min\left(1,\;\frac{D}{D_{\max}}\right)$$

where *s_d_* is a score calculated by the number of repeated detections (*D*), and *D_max_* indicates the maximum value of *D*, which is experimentally predetermined. As in the first case, when comparing these values, *s_c_* of the previously recognized parking slot is weighted by *r* (<1.0). Figure 11 depicts the parking slot tracking and combining procedure at night. In this figure, A, B, C, and D indicate the indexes of the parking slots. Figure 11a–c are the parking slot recognition results of three images captured while the vehicle is moving. As aforementioned, this method uses the constraint that one of two separating lines must be detected in the current image to reduce false detections. Thus, if only a single image is used, it is difficult to recognize the parking slots existing in the inferior quality region of the current image. This is the reason that B in Figure 11a, A in Figure 11b, and A and B in Figure 11c are unrecognized. However, if those parking slots are tracked and combined using the method described in this section, it is possible to reliably recognize all parking slots as shown in Figure 11d.

## 8. Experiments

**Figure 12:**
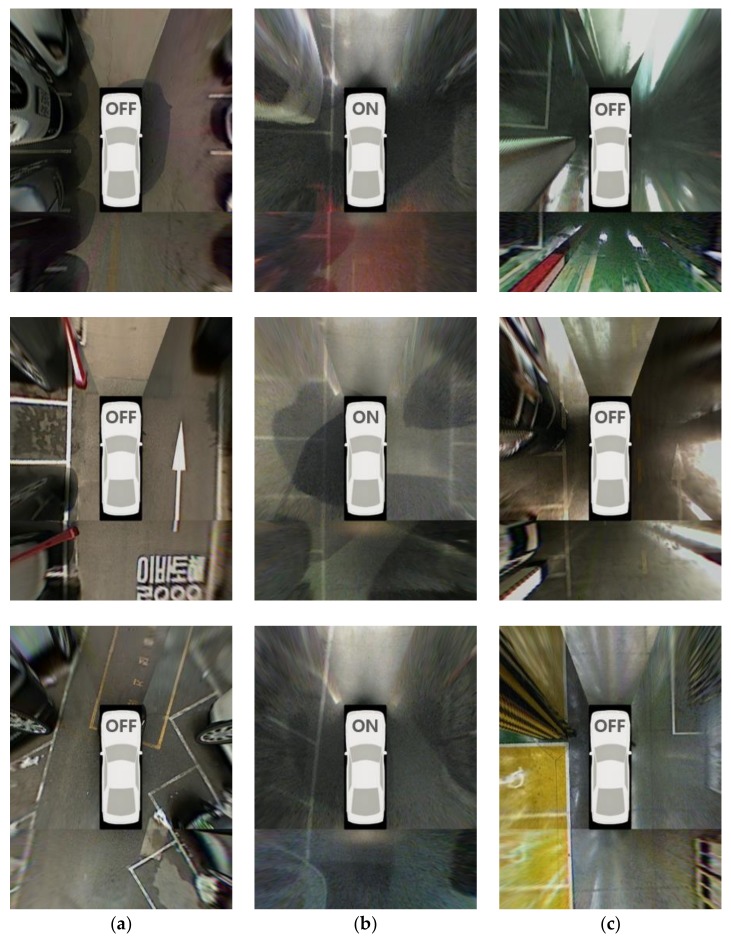
AVM images included in the test dataset. (**a**) Daytime; (**b**) Nighttime; (**c**) Underground.

The test dataset was acquired by an AVM system, ultrasonic sensors, and in-vehicle motion sensors on an off-the-shelf vehicle, the Hyundai Azera [42]. This dataset includes 609 vacant parking slots obtained in 221 different situations. Table 1 shows a detailed description of the test dataset. In terms of lighting condition, the dataset consists of 235, 214, and 160 vacant parking slots acquired during the day, at night, and underground, respectively. In terms of parking slot type, it consists of 287, 98, 120, and 104 vacant parking slots of rectangular, slanted rectangular, diamond, and open rectangular types, respectively. The outdoor conditions include all four types of parking slots, but the underground conditions include only rectangular parking slots. This is because almost all parking slots in underground parking lots are of a rectangular type in Korea owing to its visibility and space efficiency. Figure 12a–c show AVM images acquired during the day, at night, and underground, respectively. These images were selected from the test dataset. It can be seen that shadows and stains can be seen during the day, and dim lighting and reflections can be seen at night and underground.

Recall and precision in Equation (16) were used for evaluating the proposed method. These measures have also been used previously [4,5]

(16) $$\begin{array}{l} recall=\frac{number\;of\;correctly\;recognized\;slots}{number\;of\;existing\;slots}\\ precision=\frac{number\;of\;correctly\;recognized\;slots}{number\;of\;recognized\;slots} \end{array}.$$

As in previous papers [4,5], a parking slot is considered to be correctly recognized if it is recognized before the rear of the vehicle passes by it and the recognition holds until it leaves an AVM image or the vehicle stops. A parking slot is considered to be falsely recognized if it is falsely recognized more than or equal to once through an AVM image sequence.

**Figure 13:**
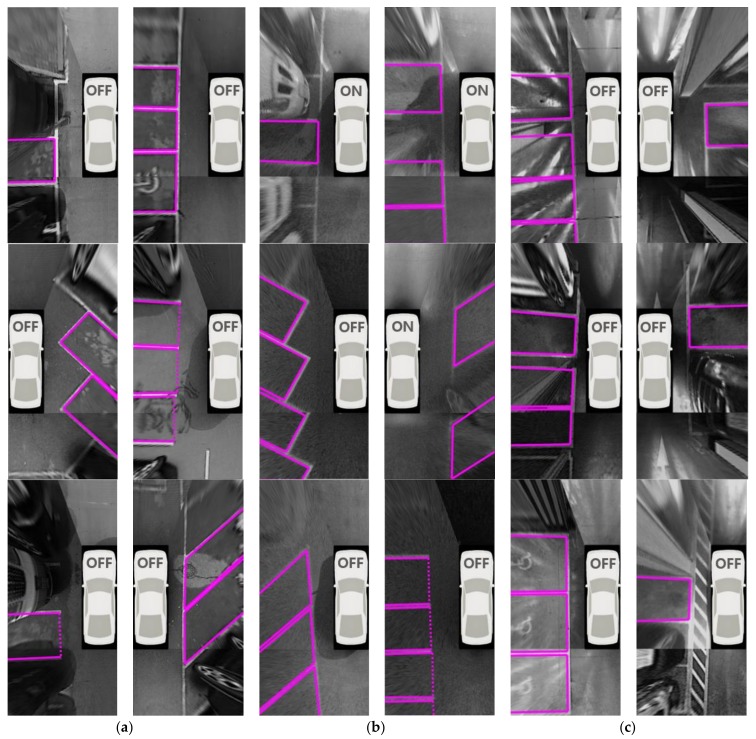
Parking slot recognition results of the proposed method. (**a**) Daytime; (**b**) Nighttime; (**c**) Underground.

**Figure 14:**
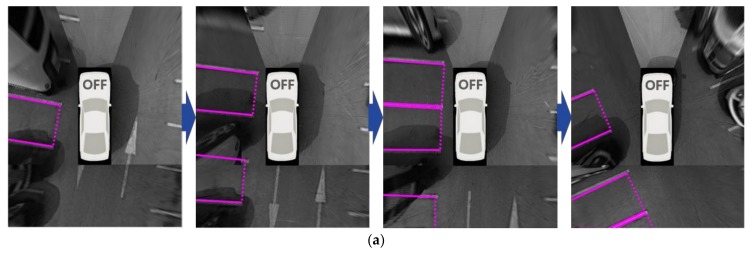

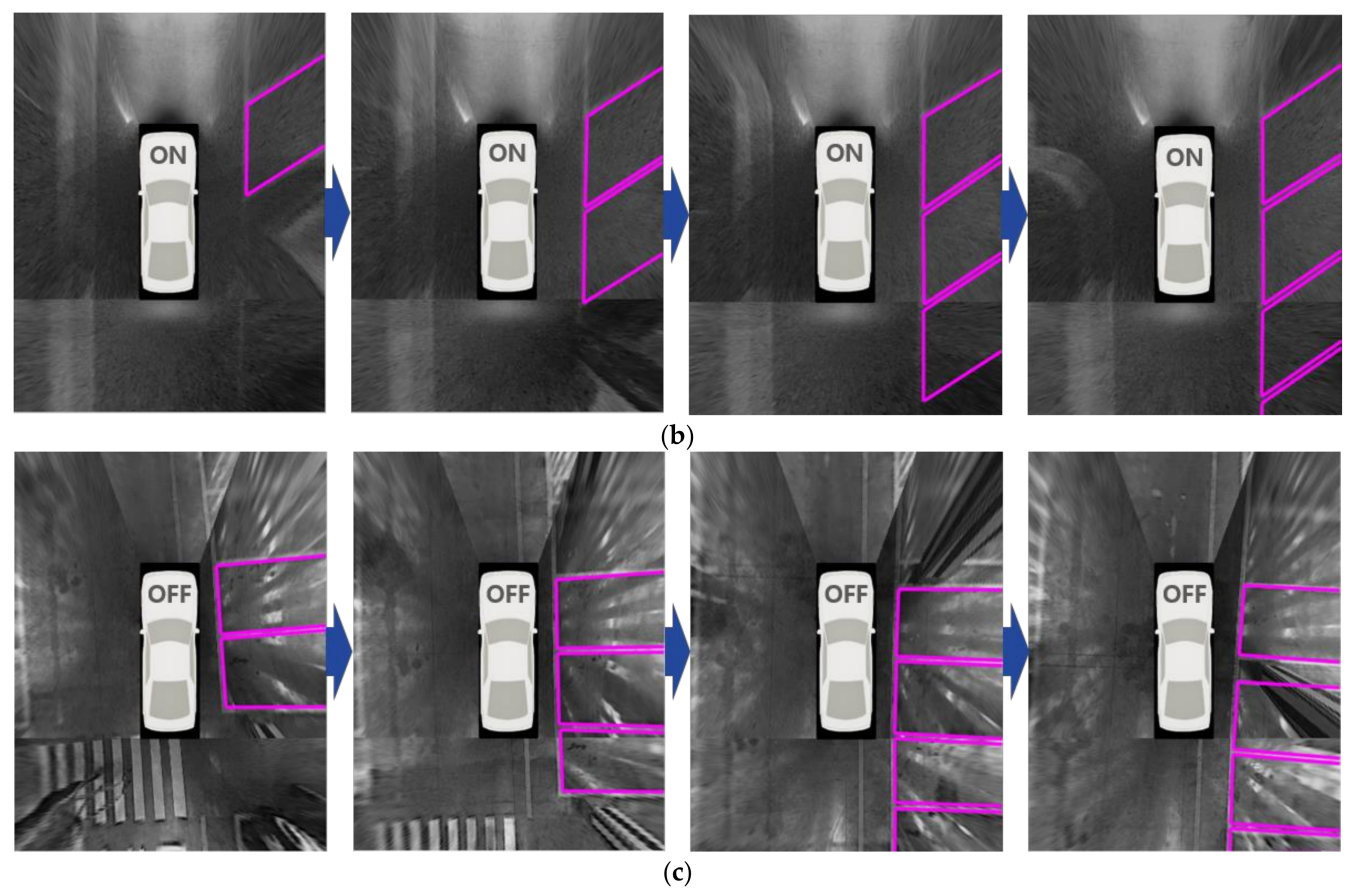
Parking slot recognition results of the proposed method in image sequences. (**a**) Daytime; (**b**) Nighttime; (**c**) Underground.

Table 2 shows the parking slot recognition performance of the proposed method. In this table, #slot, #TP, and #FP indicate the numbers of existing slots, correctly recognized slots (true positives), and falsely recognized slots (false positives), respectively. The proposed method correctly recognizes 580 parking slots out of 609 and produces 14 false positives. That is, its recall and precision are 95.24% and 97.64%, respectively. In terms of recall, this method gives the highest value (97.87%) during the day. This is because parking slot markings are clearly visible during the day because of the favorable lighting conditions. The recall of underground settings (92.50%) is lower than that of the nighttime situation (94.39%). In underground settings, both dim lighting and sever reflections on the ground hinder parking slot recognition as shown in Figure 12c, while only dim lighting hinders it in the nighttime situation. In terms of precision, the proposed method gives the highest value (99.51%) in the nighttime situation. Compared to the daytime and underground conditions, the nighttime situation includes lighting that is more severely dim, as shown in Figure 12b, and this lighting condition suppresses the edges not only from parking slot markings but also from other objects. Therefore, the proposed method rarely produces false positives in nighttime conditions. The small number of false positives means that the precision is high. In underground settings, a precision of 94.27% (the lowest precision of all settings) was achieved. This is because the edges from other objects and reflections on the ground are clearly visible, as shown in Figure 12c, so more false positives are produced in this type of setting. Figure 13 shows the final parking slot recognition results of the proposed method in various parking lot conditions. In this Figure 13a–c are the results of daytime, nighttime, and underground conditions, respectively. The entrance of the open rectangular type is depicted with a dotted line. The AVM image area with no parking slot is omitted for conciseness. The proposed method works successfully, despite the strong shadows and stains on the ground during the day (Figure 13a), the dim lighting and unclear parking slot markings at night (Figure 13b), and the strong reflections and myriad pillars underground (Figure 13c). Figure 14 shows the final parking slot recognition results of the proposed method in image sequences. These results show that the proposed method can reliably recognize various types of parking slots in a variety of lighting conditions. This is mainly because it generates parking slot candidates using separating lines, detects their entrances using line and corner features, and combines sequentially found separating lines and parking slots by tracking them using the vehicle odometry. The proposed method was implemented in C language, and its execution time was measured on a 3.40 GHz Intel Core i7-2600 CPU using a single core. It requires 54 ms/frame on average.

Since no other method can handle both various types of parking slot markings and a variety of severe lighting conditions, we carefully selected two previous methods that have different strengths and used them for performance comparison. One is the method in [4] that, among the existing methods, can recognize the largest number of parking slot marking types. The other is the method in [5] that, among the existing method, can handle the most severe lighting conditions. The method in [4] uses a hierarchical tree structure consisting of corner, junction, and slot. Table 3 shows the performance of this method in the test dataset. This method correctly recognizes 385 parking slots out of 609 and produces 239 false positives, and its recall and precision are 63.22% and 61.70%, respectively. While this method can recognize various types of parking slot markings, it is sensitive to severe lighting conditions because it uses a local feature called a corner. Since the corner feature is sensitive to amplified noise that is usually present in images captured under dim lighting, this method shows poor performance in both the nighttime and underground conditions. However, it produces a relatively good performance during the day where lighting conditions are favorable. Compared with the proposed method, this method yields a 32.02% lower recall and a 35.94% lower precision. The method in [5] uses an approach that first detects a guide line and then finds separating lines with the help of that guide line. Table 4 shows the performance of this method in the test dataset. This method correctly recognizes 251 parking slots out of 609 and produces 9 false positives, and its recall and precision are 41.22% and 96.54%, respectively. While this method is robust against unfavorable lighting conditions, it can only handle the rectangular parking slot markings. Thus, this method shows a good performance in underground settings where only rectangular parking slot markings exist. However, in terms of recall, it gives poor performances in daytime and nighttime conditions, as such settings include other types of parking slot markings. The precision of this method is quite high because it uses strong geometric constraints, which result in a small number of false positives. Compared with the proposed method, this method gives a 54.02% lower recall and a 1.10% lower precision. Table 5 summarizes Table 2, Table 3 and Table 4 by showing only the total performances of the three methods. In this table, it can clearly be confirmed that the proposed method outperforms these two other methods.

## 9. Conclusions

This paper proposes a parking slot recognition method, which is an essential component of autonomous driving. Compared with previous methods, the proposed method is more useful and practical because it can be fully automated, recognize various types of parking slot markings, and operate under unfavorable lighting conditions. To achieve these advantages, this method generates parking slot candidates using separating lines, detects their entrances using both line and corner features, and combines sequentially found separating lines and parking slots by tracking them with the vehicle odometry. Furthermore, the proposed method uses only sensors already mounted on off-the-shelf vehicles so that it can be readily commercialized. Experimental results show that the proposed method recognizes various types of parking slot markings under a variety of lighting conditions, and outperforms the previous methods.

## Figures and Tables

**Table 1:** Description of the test dataset.

Category	Type	Number of Vacant Parking Slots
Lighting condition	Daytime	235
	Nighttime	214
	Underground	160
Parking slot type	Rectangular	287
	Slanted rectangular	98
	Diamond	120
	Open rectangular	104
Total		609

**Table 2:** Parking slot recognition performance of the proposed method.

Environment	#slot	#TP	#FP	Recall	Precision
Daytime	235	230	4	97.87%	98.29%
Nighttime	214	202	1	94.39%	99.51%
Underground	160	148	9	92.50%	94.27%
**Total**	**609**	**580**	**14**	**95.24%**	**97.64%**

**Table 3:** Performance of the method in [4].

Environment	#slot	#TP	#FP	Recall	Precision
Daytime	235	219	25	93.19%	89.75%
Nighttime	214	65	161	30.37%	28.76%
Underground	160	101	53	63.13%	65.58%
**Total**	**609**	**385**	**239**	**63.22%**	**61.70%**

**Table 4:** Performance of the method in [5].

Environment	#slot	#TP	#FP	Recall	Precision
Daytime	235	65	1	27.66%	98.48%
Nighttime	214	36	0	16.82%	100.00%
Underground	160	150	8	93.75%	94.94%
**Total**	**609**	**251**	**9**	**41.22%**	**96.54%**

**Table 5:** Performance of all three methods.

Method	#slot	#TP	#FP	Recall	Precision
Proposed method	609	580	14	95.24%	97.64%
Method in [4]	609	385	239	63.22%	61.70%
Method in [5]	609	251	9	41.22%	96.54%
